# Supplementary material for: Versatile and Robust Method for Antibody Conjugation to Nanoparticles with High Targeting Efficiency
Source: Pharmaceutics. 2021 Dec 14;13(12):2153. doi: 10.3390/pharmaceutics13122153 (PMC8703776; doi:10.3390/pharmaceutics13122153)
Supplement: Supplementary file 1 [file pharmaceutics-13-02153-s001.zip › pharmaceutics-1420036-supplementary.pdf]

pharmaceutics

MDPI

Supplementary Material

Versatile and Robust Method for Antibody Conjugation to Nanoparticles with High Targeting Efficiency

Indra Van Zundert [1], Maria Bravo [1], Olivier Deschaume [2], Pierre Cybulski [1], Carmen Bartic [2], Johan Hofkens [1], Hiroshi Uji-i [1,3], Beatrice Fortuni [1,*] and Susana Rocha [1,*]

[1] Molecular Imaging and Photonics, Chemistry Department, KU Leuven, Celestijnenlaan 200F, 3001 Heverlee, Belgium; indra.vanzundert@kuleuven.be (I.V.Z.); maria.bravo@kuleuven.be (M.B.); pierre.cybulski@kuleuven.be (P.C.); johan.hofkens@kuleuven.be (J.H.); hiroshi.ujii@kuleuven.be (H.U.-I.)

[2] Department of Physics and Astronomy, Soft-Matter Physics and Biophysics Section, KU Leuven, Celestijnenlaan 200D, Box 2416, 3001 Heverlee, Belgium; olivier.deschaume@kuleuven.be (O.D.); carmen.bartic@kuleuven.be (C.B.)

[3] Research Institute for Electronic Science (RIES), Hokkaido University, N20W10, Kita Ward, Sapporo 001-0020, Hokkaido, Japan

* Correspondence: beatrice.fortuni@kuleuven.be (B.F.); susana.rocha@kuleuven.be (S.R.)

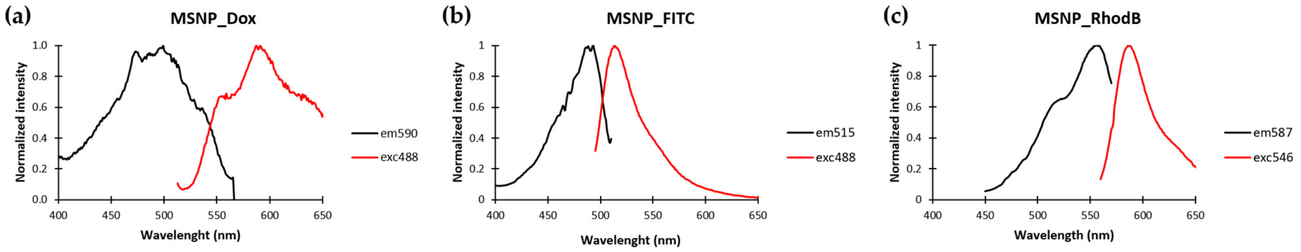

Figure S1: Emission and excitation spectra of drug and dye-loaded MSNPs. (**a**) dox-loaded MSNPs. (**b**) Fluorescein (FITC) encapsulated MSNPs and (**c**) RhodamineB (RhoB) loaded MSNPs

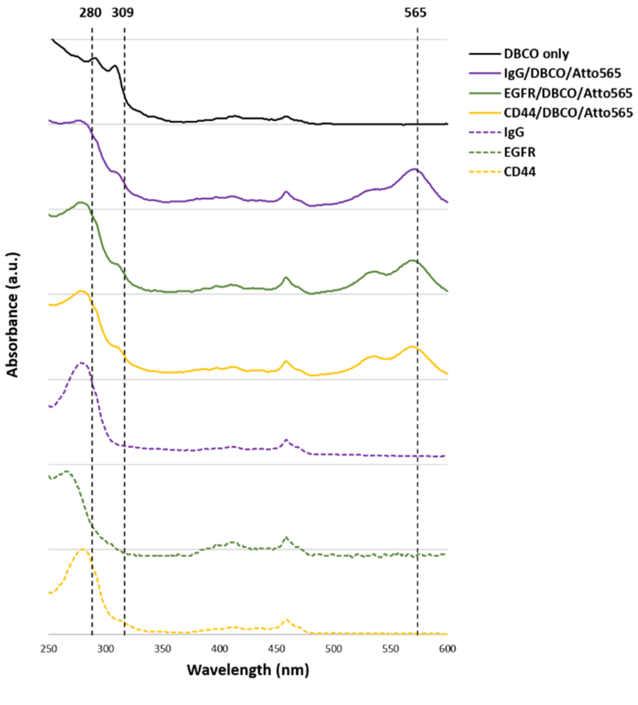

Figure S2: Absorbance for dual-labelled antibodies (IgG/DBCO/Atto565, EGFR/DBCO/Atto565 and CD44/DBCO/Atto565), non-labelled antibodies (IgG, EGFR and CD44) and DBCO only. Antibody absorption could be observed at 280 nm, a shoulder from the DBCO absorption at 309 nm and the attached dye (Atto565) at 565 nm.

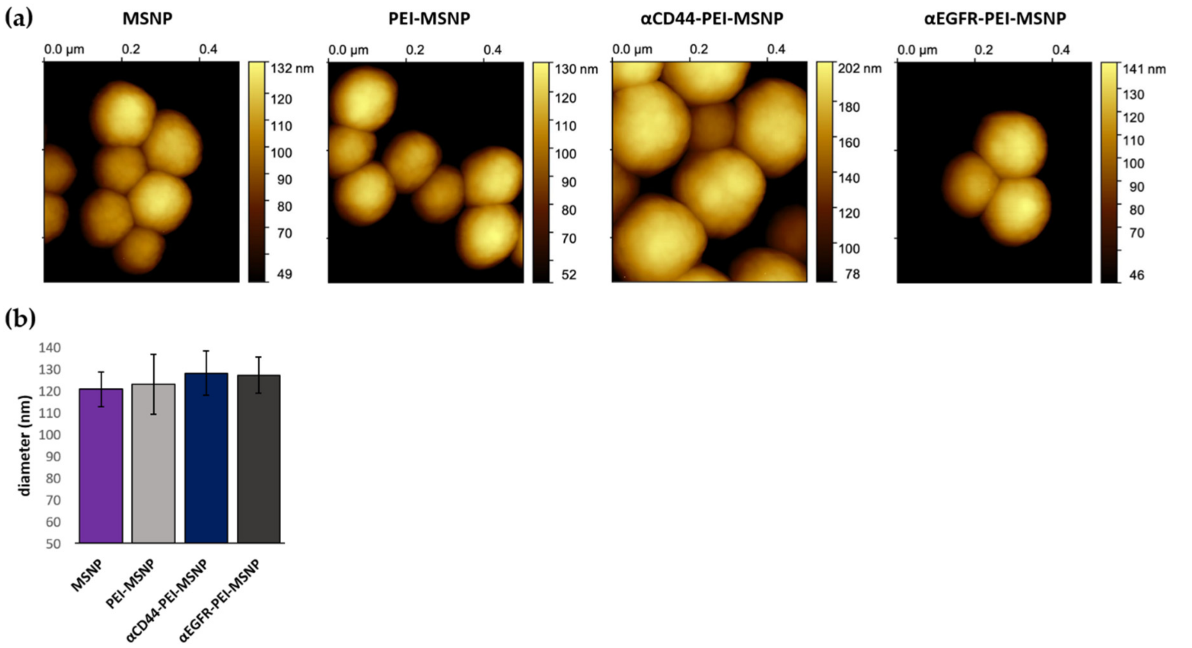

Figure S3. (**a**) AFM images of MSNPs, PEI-MSNP, αCD44-PEI-MSNPs and αCD44-PEI-MSNPs, (**b**) Graph of the average height measured with AFM (diameter). Error bars indicate ± SD.

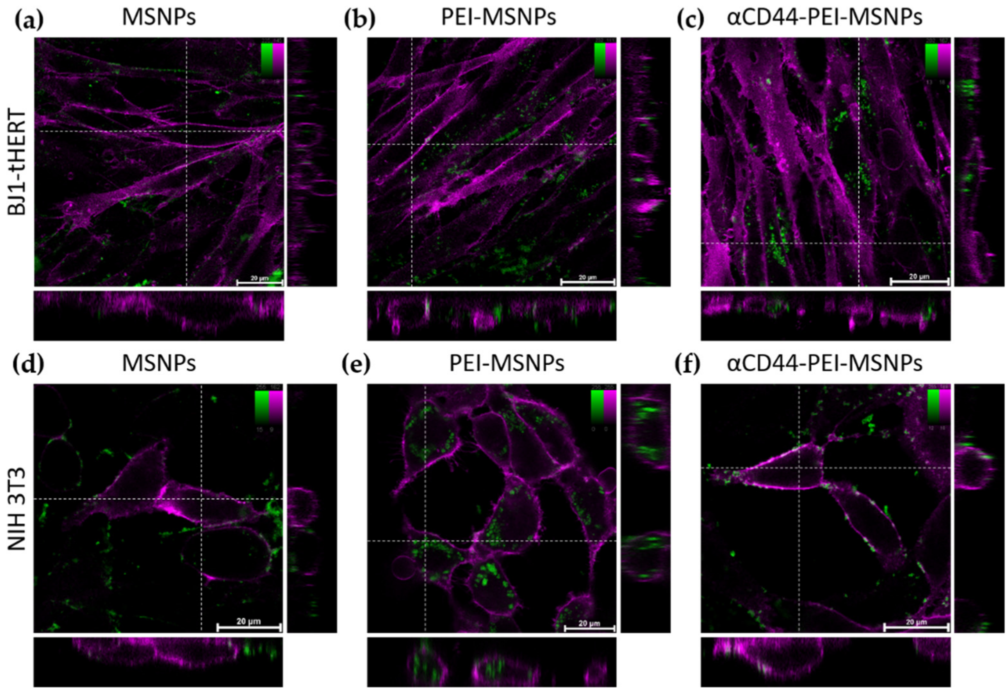

Figure S4. Confocal fluorescence microscopy images showing the influence of different MSNP coating in the uptake of nanoparticles in BJ1-tHERT and NIH 3T3 cells. Internalization of bare MSNP (**a**,**d**), PEI-coated MSNPs (PEI-MSNP, panels **b**,**e**) and CD44 functionalized MSNPs (αCD44-PEI-MSNP, panels **c**,**f**) in BJ1-tHERT cells (**a**–**c**) and NIH 3T3 cells (**d**–**f**). Nanoparticles were loaded with RhoB (green) and the plasma membrane was stained with DiR (magenta). The central square represents a single xy plane, while the bottom and left panels are the xz and yz cross-sections, indicated by the dashed lines. Scale bar is 20 µM, color bars display the intensity values.

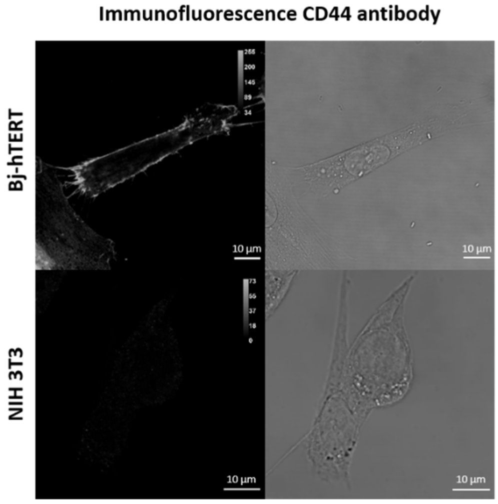

Figure S5. immunofluorescence (secondary goat-anti-rat IgG-AF488) staining showing the expression of absence of the CD44 receptor in Bj-hTERT and NIH 3T3, respectively. Scale bar is 10 µm.

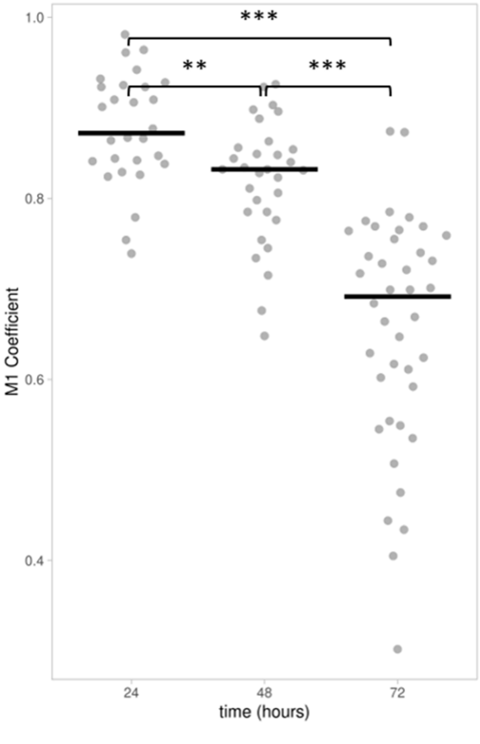

Figure S6. Manders' Coefficient (MC) representing the fraction of overlap between the αCD44-PEI-MSNPs channel with the LysoTacker Deep Red channel. n values for 24, 48 and 72 h are 28, 31 and 40, respectively. ** ($p < 0{,}01$) and *** ($p < 0{,}001$) .

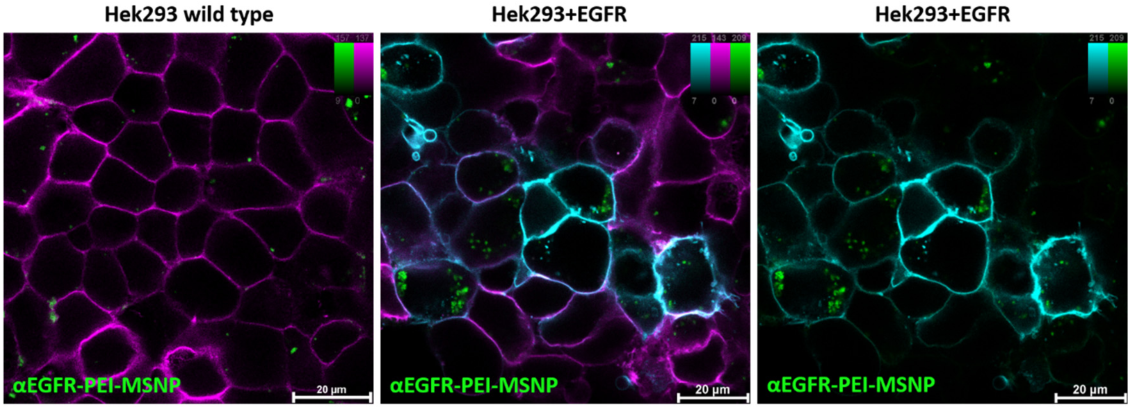

Figure S7. Internalization of αEGFR-PEI-MSNPs in wild type Hek293 (left panel) and Hek293 cells transiently transfected with a pcDBA3-EGFR-HaloTag® (middle and right panel). The right panel shows the nanoparticle and EGFR receptor channel overlay. The EGFR receptors in the transfected cells (Hek293+EGFR), after staining with a 488 HaloTag® ligand, are shown in cyan. αEGFR-PEI-MSNPs, encapsulated with FITC, are depicted in green. The cells membrane, stained with DiR, is colored magenta.

Table S1. αCD44-PEI-MSNP aggregation in different solutions: average OD600 values for αCD44-PEI-MSNPs dissolved in MilliQ water, FBS and DMEM with 10% FBS before and after 24 hours of incubation at 37 °C . Mean ± SD (3 measurements in 3 different days).

	**0 h**	**24 h**
**MilliQ water**	0.09 ± 0.006	0.10 ± 0.006
**FBS**	0.10 ± 0.005	0.10 ± 0.006
**DMEM+10%FBS**	0.13 ± 0.01	0.12 ± 0.017
